# RSNET: inferring gene regulatory networks by a redundancy silencing and network enhancement technique

**DOI:** 10.1186/s12859-022-04696-w

**Published:** 2022-05-06

**Authors:** Xiaohan Jiang, Xiujun Zhang

**Affiliations:** 1 Key Laboratory of Plant Germplasm Enhancement and Specialty Agriculture, Wuhan Botanical Garden, Chinese Academy of Sciences, Wuhan, 430074 China; 2 Center of Economic Botany, Core Botanical Gardens, Chinese Academy of Sciences, Wuhan, 430074 China; 3 University of Chinese Academy of Sciences, Beijing, 100049 China

**Keywords:** Gene regulatory network, Indirect interaction, Network inference, Network enhancement, Redundancy silencing

## Abstract

**Background:**

Current gene regulatory network (GRN) inference methods are notorious for a great number of indirect interactions hidden in the predictions. Filtering out the indirect interactions from direct ones remains an important challenge in the reconstruction of GRNs. To address this issue, we developed a redundancy silencing and network enhancement technique (RSNET) for inferring GRNs.

**Results:**

To assess the performance of RSNET method, we implemented the experiments on several gold-standard networks by using simulation study, DREAM challenge dataset and *Escherichia coli* network. The results show that RSNET method performed better than the compared methods in sensitivity and accuracy. As a case of study, we used RSNET to construct functional GRN for apple fruit ripening from gene expression data.

**Conclusions:**

In the proposed method, the redundant interactions including weak and indirect connections are silenced by recursive optimization adaptively, and the highly dependent nodes are constrained in the model to keep the real interactions. This study provides a useful tool for inferring clean networks.

**Supplementary Information:**

The online version contains supplementary material available at 10.1186/s12859-022-04696-w.

## Background

Gene regulatory network (GRN), which represents interactions or causalities between genes, describes the developmental or regulatory process in a cellular system [1]. GRN inference is a focal point of systems biology to understand biological systems [2]. The traditional knock-out or perturbation experiments have been widely used to discover the regulations among genes and achieved success in some degree to understand the biological system [3]. However, these interactions discovered by the expensive and time-consuming experiments are 'just the tip of the iceberg' in a complex GRN. While the genome-wide inference of GRNs from high-throughput data by computational methods promises an economical channel to disclose the complex regulatory mechanism [4, 5]. The challenge of computational methods is to build reasonable models to precisely predict the interactions between regulators and targets from gene expression data [6]. Distinguishing the direct interactions from the indirect ones remains an important challenge in the reconstruction of GRNs because of the notoriousness of the inference methods with the indirect interactions inherited in the network [7, 8].

In recent years, various approaches have been developed to address these challenges in GRN inference, and some of them have achieved success in some degree [9]. According to the techniques involved, these approaches can be divided into two types, i.e., dependence and equation-based methods [10]. In dependence-based methods, gene network is predicted by measuring the dependences among genes based on the methods such as *Pearson* correlation coefficient [11–13], mutual information [14, 15], and *Granger* method [16, 17]. This types of methods can measure the linear or nonlinear correlations independently but the results involve lots of redundant edges like indirect regulations [18–20]. In equation-based methods, the regulations and regulatory strengths among genes are described as equations [21]. Representative equation-based methods contain multiple linear regression [22], nonnegative matrix factorization [23], network component analysis [24, 25], and linear programming [26], and random forest [27, 28]. The equation-based methods can catch the interactions based on the dynamic mechanism but the optimization technique sometimes impacts their capability of parameter estimation for the high dimensionality of candidate regulators [29, 30].

Despite concurrent advances in GRN inference methods, most of them cannot distinguish direct correlations from the indirect ones [31]. Some dependence-based methods have been developed to discriminate direct and indirect connections of GRNs, such as partial correlation coefficient (PCC) [32], conditional mutual information (CMI) [33], part mutual information (PMI) [34], and conditional mutual inclusive information (CMI2) [35]. The equation-based methods are popular for their advantages of sparseness control and optimal estimation [36–38]. However, these methods are sensitive to the data with tow limitations which impact the performance of GRN inference seriously [39, 40]. Firstly, the noise of the data, high dimensionality of genes, and small scale of samples will affect parameter estimation of optimization. Secondly, indirect interactions will be involved in the results [41, 42]. The challenge to improve the accuracy of regression-based methods is to address these limitations [43, 44].

We previously proposed a noise and redundancy reduction strategy, namely NARROMI, based on recursive optimization that improved the performance on gene network inference [45]. In this strategy, the network was updated by recursive optimization to remove the indirect interactions. The limitation of the strategy is that some direct interactions identified by previous step were not recognized by next step. In other words, accompanied with the elevated true positive rate (TPR), recursive optimization (RO) also improves false negative rate (FPR). In an algorithm for network inference, the balance between TPR and FPR is the key technique to improve its performance. Some techniques incorporating existing network information into the optimization problem have been proposed to improve network inference [46, 47].

To reduce FPR and improve TPR simultaneously in one model, we developed a redundancy silencing and network enhancement technique (RSNET) for inferring GRNs. In the proposed method, the redundant interactions are silenced by significant MI firstly and then the recursive optimizations based on the updated results. In the meanwhile, the candidate genes with highly dependent parameters measured from the data by mutual information (MI) are constrained in the model as network enhancement items. In the process of the algorithm, the noisy regulators will be filtered out by measuring the correlations between regulators, the highly putative candidate regulators will be constrained as supervisors to improve the efficiency of optimization, and the indirect nodes will be filtered out by the recursive optimizations step by step. To assess the performance of RSNET method, we implemented the experiments on several gold-standard networks by using simulation study, DREAM challenge dataset and *Escherichia coli* network. The results show that RSNET method performed better than the compared methods in sensitivity and accuracy. As a case of study, RSNET was used to infer the functional GRN for fruit development from gene expression data in apple. RSNET software is freely accessible at https://github.com/zhanglab-wbgcas/rsnet.

## Results

### RSNET algorithm

**Fig. 1:**
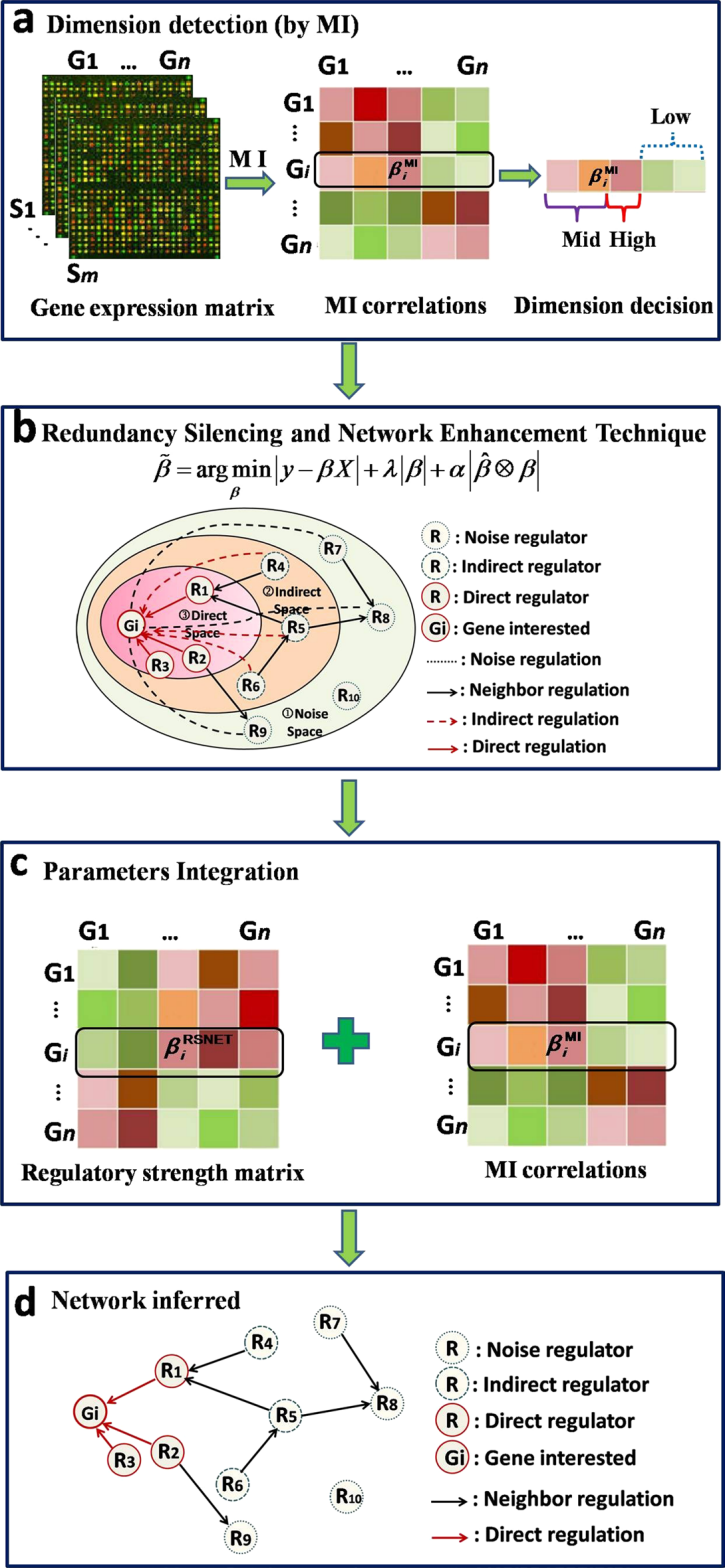
The flowchart of RSNET method. **a** The initial dimensionality is detected by using MI measure. The candidate genes will be separated to three classes, i.e. low-dependent, mid-dependent and high-dependent genes. **b** An example for the prediction. The regulatory spaces include three types, i.e. direct space, indirect space and noise space. There are four types of interactions, i.e. direct regulation, indirect regulation, noise regulation and neighbour regulation. **c** The regulatory strengths are estimated by combining MI measures and RSNET parameters. **d** The network inferred by RSNET. The final network excluded the noise and direct regulations

To accurately infer the underlying direct GRNs from the expression data, we develop a novel technique, i.e. redundancy silencing and network enhancement technique (RSNET). Figure 1 provides the flowchart of RSNET method.

As shown in Fig. 1a, we use the MI measure to decide a small but not biased searching space. With the different thresholds, we divide the candidate genes into three classes, i.e. the low dependent or independent, mid-dependent and high-dependent genes. Omitting the low-dependent genes, we get the initial dimensionality of the regression model. With the other two classes of genes, i.e. mid-dependent and high-dependent genes, we estimate the regression parameters as regulatory strengths. In addition, we use the high-dependent genes for the network enhancement items in the regression model, i.e. the high-dependent genes will be constrained in the model.

For each target gene, we use the constraint-based recursive optimization model for the estimation of regulatory strengths. We use the high-dependent genes as the network enhancement items in the regression model. This will induce more accurate estimation of parameters than the standard regression model. These network enhancement items have the priority to be kept in the result than other general genes because of the constrain technique. In the meanwhile, we filter out the indirect regulators gradually by RSNET algorithm.

Figure 1b shows the core procedure of RSNET method. According to the type of regulators, we divided the regulatory space into three spaces, i.e. direct space, indirect space and noise space. For a given interested gene, there are three types of regulators, i.e. direct regulator, indirect regulator and noise regulator. There are four types of interactions, i.e. direct regulation, indirect regulation, noise regulation and neighbour regulation. In this algorithm, we will keep direct and neighbour regulations in the last prediction as real interactions, but filter out indirect and noise regulations to improve the prediction. Figure 1b provides an example for the prediction. In the prediction, **G*i*** is a given interested gene and **R*j*** (*j* = 1,2,…,10) are ten candidate genes in three regulatory spaces. **R1**, **R2** and **R3** are three direct/real regulators in direct space. **R4**, **R5** and **R6** are three indirect regulators in indirect space. **R7**, **R8**, **R9** and **R10** are four noise regulators in noise space. For the interested gene **G*i***, we will filter out noisy and indirect regulators **R4-R10** in the result.

To combine linear and nonlinear interactions between regulators and targets, we estimate the regulatory strengths by combining MI measures and RSNET parameters with balance parameter (Fig. 1c). As shown in Fig. 1d, we construct the network by the combined regulatory strengths. In the final network, we exclude noise and indirect regulations. As real regulations of neighbour regulators, we keep the neighbour regulations in the final network.

### Simulation study

To evaluate the performance of RSNET method, the simulation study was implemented by using synthetic gene network and expression data. In this study, six networks with sizes 10, 50, 100, 500, 1000 and 5000 as well as matched expression data with samples 5, 7, 10, 15, 20 and 25 respectively were generated. The expression noise with 10 percentages was randomly imbedded during the data simulation. In the experiment, our RSNET method was compared with methods LASSO, LP, RO, ARACNE, GENIE3 and NARROMI.

**Fig. 2:**
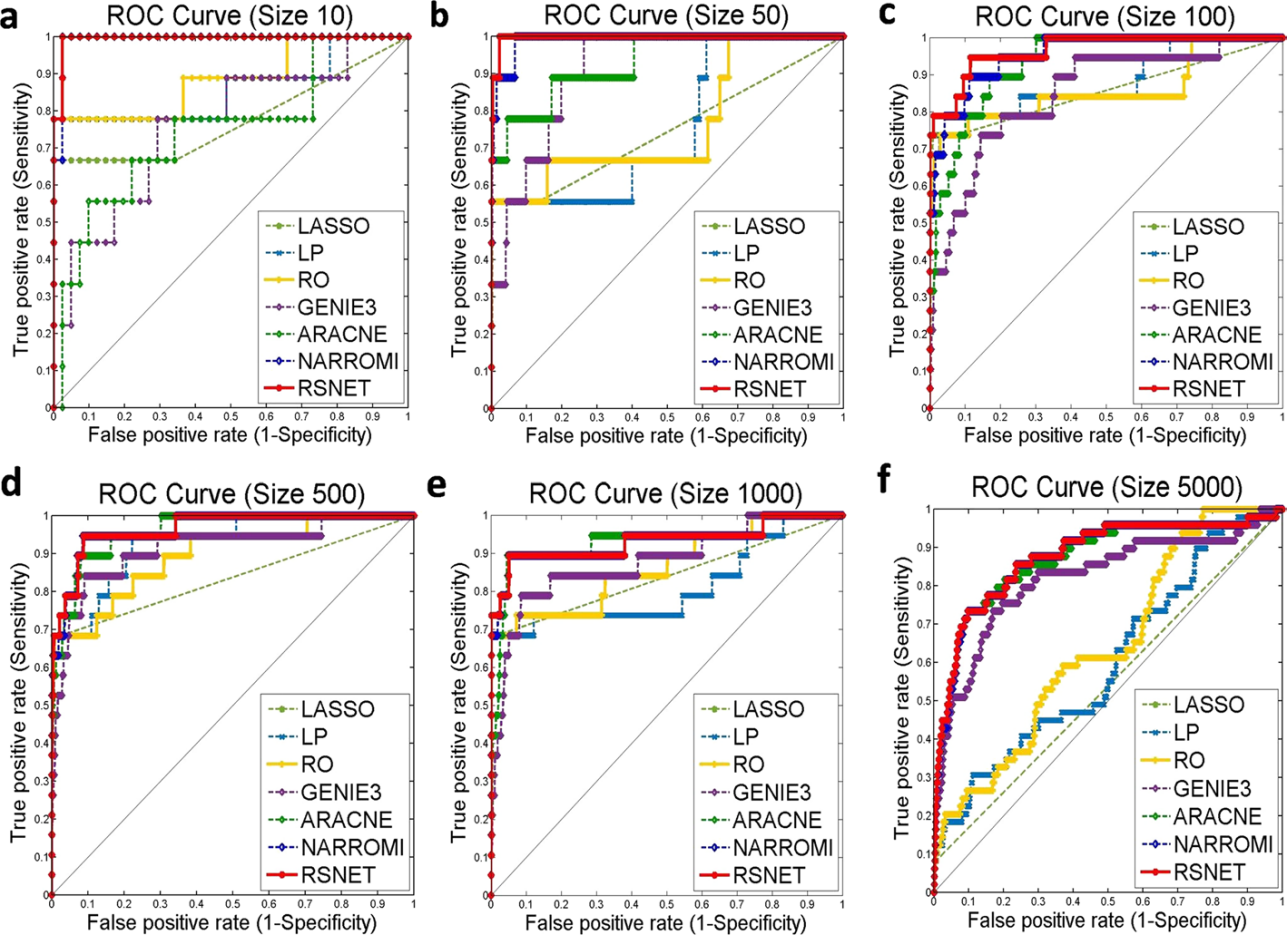
The ROC curves on synthetic dataset. **a** Scale 10. **b** Scale 50. **c** Scale 100. **d** Scale 500. **e** Scale 1000. **f** Scale 5000

The results on benchmark networks with different were described in Fig. 2 with the receiver operating characteristic (ROC) curves. Our RSNET method performed better than other four methods with highest ROC curves which were plotted with red lines in Fig. 2a–f. To describe the performance metrics in detail, Table 1 listed the performance indices for these compared methods. RSNET method performed best on all the three dataset with AUC values 0.9946, 0.9968, 0.9668, 0.9661, 0.9325 and 0.8770. When the network size is more than 1000, the accuracy of RSNET is still high enough. We conclude that the network scale affects the performance of RSNET method very few. The results indicate that RSNET method improves network inference by silencing the redundant edges. In addition, we also computed the running time of RSNET method on networks with different sizes. The CPU times for networks with sizes 10, 50, 100, 500, 1000, 5000 were 0.0889, 1.0716, 3.4185, 43.9768, 164.0785 and 4059.2665 s. From the results, we can conclude that the RSNET is an efficient and time-saving method for network inference.

**Table 1:** The results on synthetic networks with scales 10, 50,100, 500, 1000 and 5000

Approach	FPR	TPR	ACC	PPV	MCC	AUC
*Scale 10*						
LASSO	0.1220	0.6667	0.8400	0.5455	0.5052	0.7764
LP	0.0243	0.7778	0.9400	0.8750	0.7895	0.8591
RO	0.0731	0.7778	0.9000	0.7000	0.6768	0.8862
GENIE3	0.1707	0.4444	0.7600	0.3636	0.2539	0.7317
ARACNE	0.1463	0.5556	0.8000	0.4545	0.3795	0.7480
NARROMI	0.0243	1.0000	0.9800	0.9000	0.9370	0.9919
**RSNET**	**0.0000**	**0.8889**	**0.9800**	**1.0000**	**0.9315**	**0.9946**
*Scale 50*						
LASSO	0.00611	0.5556	0.9860	0.6250	0.5822	0.7484
LP	0.00203	0.5556	0.9900	0.8333	0.6759	0.7579
RO	0.01222	0.5556	0.9800	0.4545	0.4925	0.7669
GENIE3	0.02037	0.3333	0.9680	0.2308	0.2615	0.9131
ARACNE	0.07536	0.7778	0.9220	0.1591	0.3296	0.9301
NARROMI	0.01222	0.6667	0.9820	0.5000	0.5685	0.9898
**RSNET**	**0.01222**	**0.7778**	**0.9840**	**0.5385**	**0.6396**	**0.9968**
*Scale 100*						
LASSO	0.0081	0.7368	0.9870	0.6364	0.6782	0.8576
LP	0.0101	0.6316	0.9830	0.5455	0.5784	0.8795
RO	0.0101	0.6316	0.9830	0.5455	0.5784	0.8596
GENIE3	0.0193	0.3158	0.9680	0.2400	0.2592	0.8903
ARACNE	0.0428	0.5789	0.9500	0.2075	0.3267	0.9323
NARROMI	0.0101	0.6842	0.9840	0.5652	0.6139	0.9553
**RSNET**	**0.0050**	**0.7895**	**0.9910**	**0.7500**	**0.7649**	**0.9668**
*Scale 500*						
LASSO	0.0018	0.5789	0.9966	0.5500	0.5626	0.8373
LP	0.0018	0.5789	0.9966	0.5500	0.5626	0.9291
RO	0.0022	0.5789	0.9962	0.5000	0.5361	0.8986
GENIE3	0.0140	0.4737	0.9940	0.1139	0.2268	0.9340
ARACNE	0.0072	0.5789	0.9912	0.2340	0.3645	0.9598
NARROMI	0.0024	0.5789	0.9960	0.4783	0.5242	0.9652
**RSNET**	**0.0030**	**0.5789**	**0.9954**	**0.4231**	**0.4927**	**0.9661**
*Scale 1000*						
LASSO	0.0006	0.6316	0.9987	0.6667	0.6482	0.8392
LP	0.0008	0.6842	0.9986	0.6190	0.6501	0.8124
RO	0.0007	0.6842	0.9987	0.6500	0.6662	0.8665
GENIE3	0.0008	0.3158	0.9915	0.0657	0.1401	0.8844
ARACNE	0.0006	0.2105	0.9979	0.4000	0.2892	0.9296
NARROMI	0.0007	0.6842	0.9987	0.6500	0.6662	0.9316
**RSNET**	**0.0009**	**0.7368**	**0.9986**	**0.6087**	**0.6690**	**0.9325**
*Scale 5000*						
LASSO	0.00058	0.0204	0.9985	0.0333	0.0253	0.5386
LP	0.00058	0.0204	0.9985	0.0333	0.0253	0.5998
RO	0.00092	0.0612	0.9982	0.0612	0.0603	0.6418
GENIE3	0.00605	0.1840	0.9932	0.0289	0.0331	0.8322
ARACNE	0.00034	0.0204	0.9987	0.0556	0.0707	0.8719
NARROMI	0.00102	0.0612	0.9981	0.0556	0.0574	0.8754
**RSNET**	**0.00116**	**0.0612**	**0.9979**	**0.0492**	**0.0538**	**0.8770**

### Performance on DREAM challenge networks

**Fig. 3:**
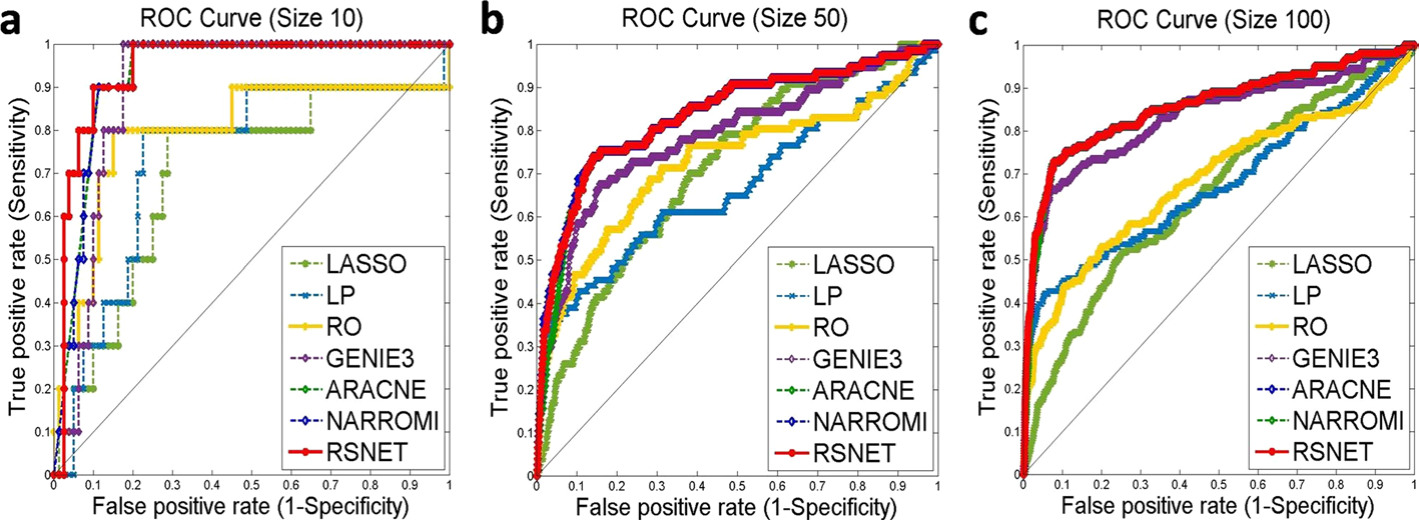
The ROC curves on DREAM challenge networks. **a** Scale 10. **b** Scale 50. **c** Scale 100

To evaluate the method, we also implemented the study on the benchmark gene networks and expression data from DREAM challenge. The gene expression data were simulated based on *Yeast* and *E.coli* gene regulatory networks that were experimentally confirmed [48]. The datasets include 3 *Yeast* and 2 *E. coli* networks with scales 10, 50 and 100 [7]. The ROC curves for the compared methods on these datasets are provided in Fig. 3.

Firstly, the experiment on small-scale network (*Yeast* 1) with 10 genes was implemented. The threshold value for the parameter of low-regulations was set to 0.05, and the threshold value for the parameter of high-regulations was set to 0.2. RSNET method re-estimated regulatory strengths based on the result of the former computation until there was no change in network structure. The ROC curves of these compared methods are described in Fig. 3a. The results show that RSNET method performs the best among the compared methods. The AUC curve in red is for RSNET method and its AUC score reaches 0.945. Table 2 (Scale 10) provides the indices values such as TPR, FPR, PPV, etc. The indices values show that RSNET method do better that compared methods. This indicates that RSNET method can remove the redundant edges by redundancy silencing and network enhancement strategy.

**Table 2:** The results on DREAM networks with scales 10, 50 and 100

Approach	FPR	TPR	ACC	PPV	MCC	AUC
*Scale 10*						
LASSO	0.8375	0.6000	0.2111	0.0822	− 0.1907	0.7025
LP	0.4125	0.1000	0.5333	0.0294	− 0.2026	0.7388
RO	0.5000	0.1000	0.4556	0.0244	− 0.2524	0.7975
GENIE3	0.1125	0.7000	0.8889	0.5000	0.6187	0.9212
ARACNE	0.1125	0.9000	0.8889	0.5000	0.6187	0.9300
NARROMI	0.1125	0.9000	0.8889	0.5000	0.6187	0.9294
**RSNET**	**0.0375**	**0.7000**	**0.9333**	**0.7000**	**0.6625**	**0.9450**
*Scale 50*						
LASSO	0.1285	0.3506	0.8551	0.0813	0.1132	0.7110
LP	0.0847	0.3896	0.8988	0.1299	0.1820	0.6686
RO	0.1311	0.4935	0.8571	0.1089	0.1809	0.7268
GENIE3	0.0745	0.4805	0.9114	0.1729	0.2508	0.8004
ARACNE	0.0817	0.5974	0.9082	0.1917	0.3027	0.8325
NARROMI	0.0623	0.5325	0.9249	0.2169	0.3074	0.8389
**RSNET**	**0.0594**	**0.5195**	**0.9273**	**0.2210**	**0.3069**	**0.8376**
*Scale 100*						
LASSO	0.0510	0.1807	0.9361	0.0569	0.0741	0.6536
LP	0.0462	0.4036	0.9445	0.1296	0.2063	0.6741
RO	0.0854	0.3735	0.9055	0.0693	0.1290	0.6856
GENIE3	0.0255	0.4096	0.9657	0.2315	0.3076	0.8407
ARACNE	0.0330	0.5060	0.9592	0.2069	0.3062	0.8572
NARROMI	0.0243	0.4639	0.9671	0.2452	0.3220	0.8584
**RSNET**	**0.0259**	**0.5120**	**0.9663**	**0.2515**	**0.3437**	**0.8594**

Secondly, we evaluated the methods on the network (*Yeast* 1) with scale 50. For RSNET method, the threshold value for the low-regulation parameters was set to 0.05, and the threshold value for high-regulation parameter was set to 0.2. As a result of RSNET method, AUC score is 0.838. It performed best among the compared methods (Fig. 3b**)**. TPR and FPR values showed that RSNET method outperformed other methods obviously (Scale 50, Table 2). With the scores 0.0594, 0.9273, 0.2210 and 0.3069, FPR, ACC, PPV and MCC proved the good performance of RSNET method. In this experiment, RSNET method successfully silenced the redundant edges over-estimated by previous methods.

Lastly, we evaluated RSNET method on network (*Yeast* 1) with scale 100. There are 166 links in the gold-standard network. The threshold for low-regulation parameters was set to 0.03 and the threshold for high-regulation parameter was set to vale 0.1. As the result of the experiment, Fig. 3c described the AUC curves of these methods. AUC score for RSNET method is 0.8594 which is greater than AUC scores of other methods. In this experiment, RSNET method got the highest scores in MCC, ACC, PPV and TPR, i.e. 0.3437, 0.9663, 0.2515 and 0.5120 (Scale 100, Table 2). RSNET method successfully silenced the redundant edges over-estimated by LP method and improved the TPR value from 0.1870 to 0.5120. The AUC value was improved by the redundancy silencing and network enhancement strategy from 0.6536 for LASSO method to 0.8594 for RSNET method.

The comparison results for networks *Yeast* 2, *Yeast* 3, *E.coli* 1 and *E.coli* 2 were provided in Additional file 1: Table S1 which shows the good performance of RSNET on network inference. The results above proved the efficiency of RSNET method on DREAM gene network inference. As a new technique by redundancy silencing and network enhancement, RSNET proved itself a perfect direct interactions estimation technique.

### Performance on real gene network

To evaluate the method by using real gene expression data to reconstruct gene network, we collected the benchmark network from the *Escherichia coli* network database [49] and gene expression data from *Escherichia coli* data bank [50]. As a result of the data processing, a network with 160 TFs and 1258 genes are generated. There are 2765 links among these genes in this benchmark network. The network degree of the benchmark network is around 2. To measure the performance of the compared method, the AUC scores for regulatory strengths of the candidate TFs on a given target gene (TG) and the AUC scores for regulatory strengths of a given TF on all the putative target genes were computed because the network size is too big. For the calculated AUCs, the box plot with minimum, maximum, median, and mean values was drawn. In addition, the numbers as well as percentages of TGs or TFs with more than certain AUC values were calculated.

**Fig. 4:**
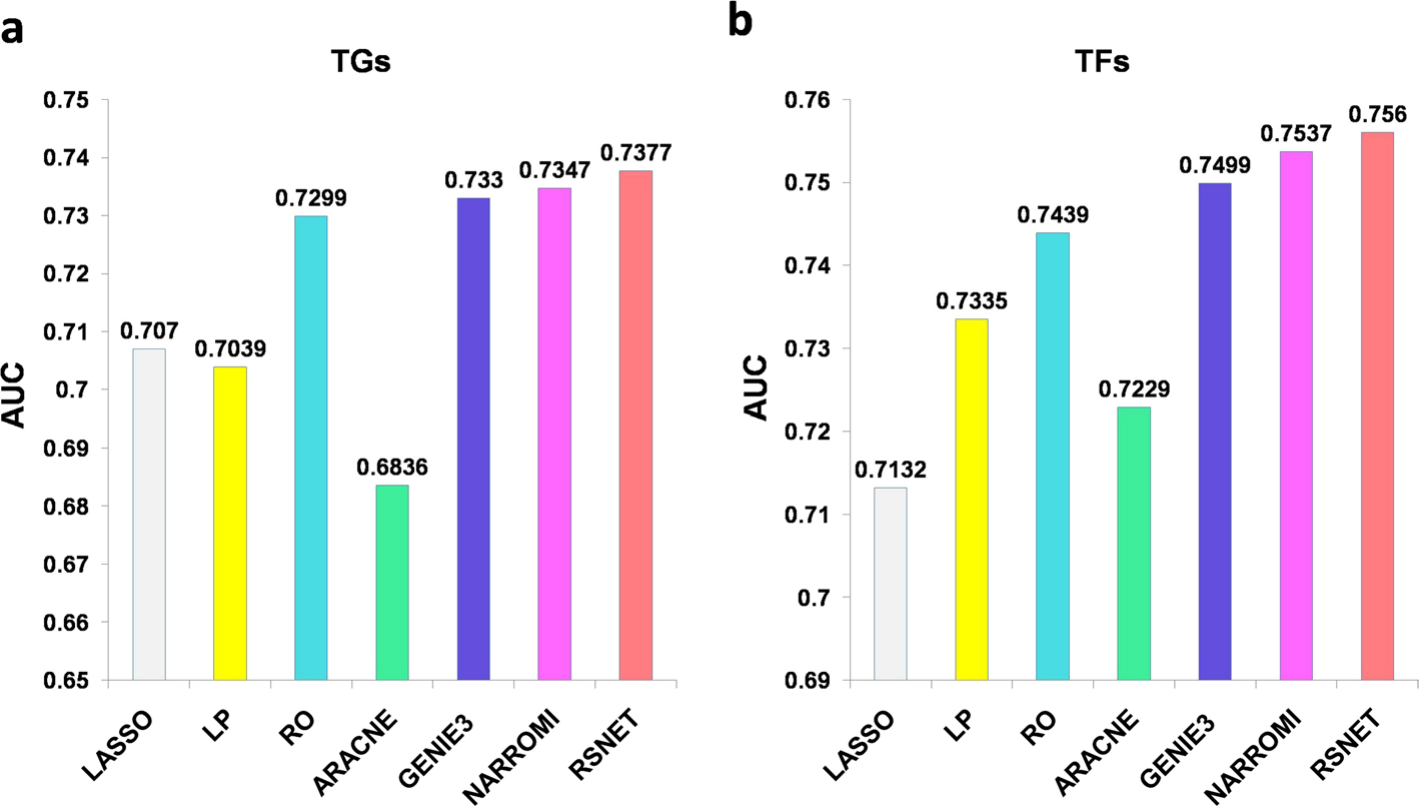
The AUC statistics for different methods. **a** Box plots for target genes (TGs). **b** Box plots for TFs

**Fig. 5:**
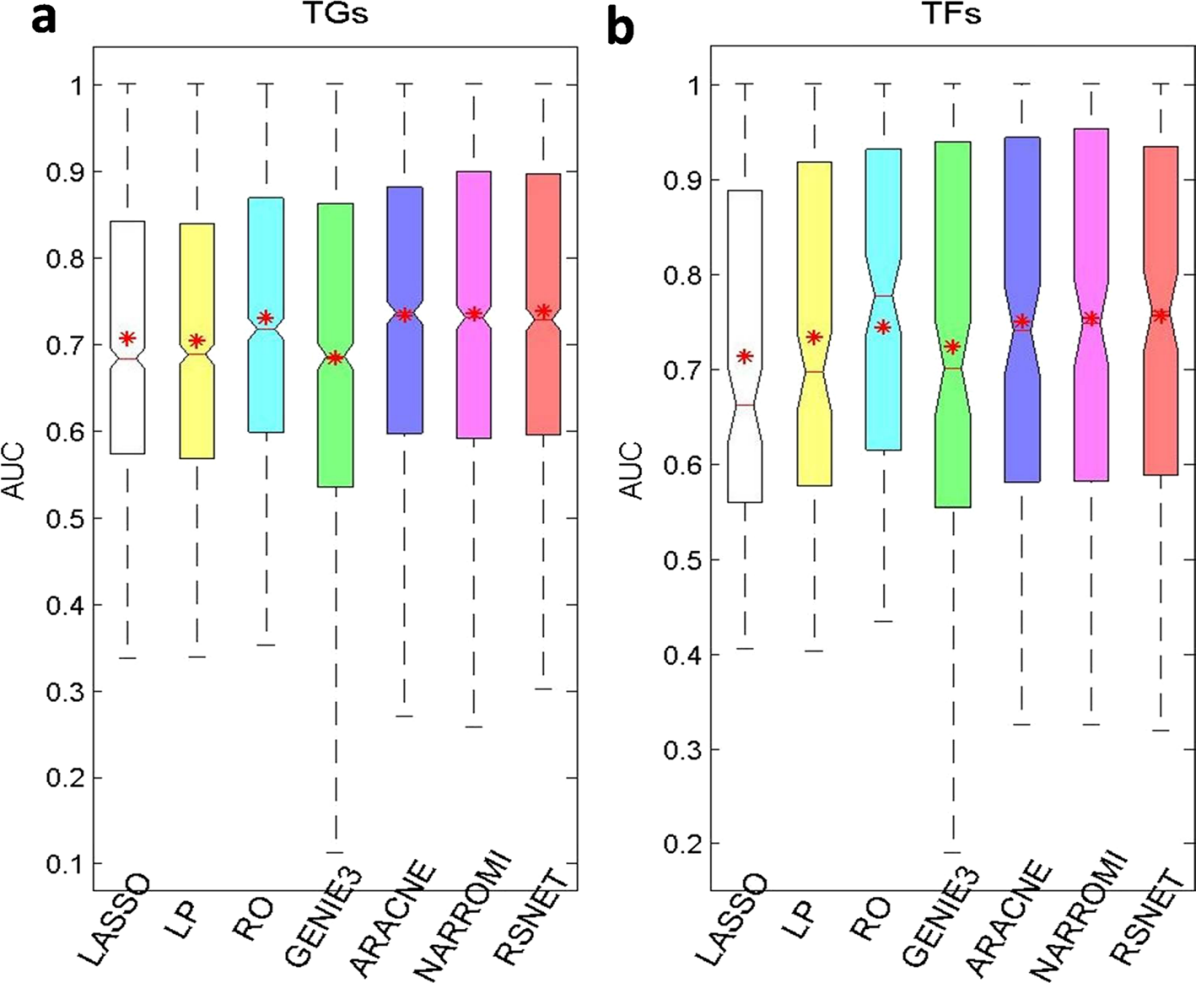
The comparison of global AUC values for different methods. **a** The global AUCs for all the target genes. **b** The global AUCs for all TFs

Figure 4a is the box plot for the AUCs of the target genes (TGs). We can find that RSNET outperforms other three methods in maximum, median and mean AUC values. Figure 4b is the box plot for the AUCs of the TFs. The result shows that RSNET performed the best on minimum, median and mean AUC values. Figure 5a is the global/average AUCs for all the TGs and Fig. 5b is the global/average AUCs for all the TFs. All the results show that RSNET method performs better than other compared methods. Table 3 provides average AUCs for TGs (or TFs) and the number of TGs (or TFs) with the AUC values higher than 0.8. All these results show that RSNET method performed the best among the compared methods.

**Table 3:** The AUC scores for compared methods on real *Escherichia coli* network

Approach	LASSO	LP	RO	GENIE3	ARACNE	NARROMI	**RSNET**
AveAUC (TG)	0.7070	0.7039	0.7299	0.6836	0.7330	0.7347	**0.7377**
AUC > 0.8 (TG)	384 (30.52)	367 (29.17)	439 (34.89)	428 (34.02)	484 (38.47)	485 (38.55)	**490 (38.95)**
AveAUC (TF)	0.7132	0.7335	0.7439	0.7229	0.7499	0.7537	**0.7560**
AUC > 0.8 (TF)	57 (36.77)	58 (37.41)	71 (45.80)	60 (38.70)	68 (43.87)	71 (45.80)	**72 (46.45)**

AveAUC*,* Average of AUCs for TG/TF; AUV > 0.8*,* the number of TGs/TFs; '()'*,* the numbers in brackets are percentages

### Identification of apple fruit development-specific network

In plant, the phenotype is decided by a certain functional gene network. As one of the most important phenotype, fruit development has become the research topic on fruit crop [51, 52]. As a case study, RSNET method was used to predict fruit development-specific gene regulatory network in *apple* (Malus domestica 'Royal Gala') from gene expression data. In the dataset, there are eight time-point samples from floral bud to ripe fruit during fruit development [53]. From the original gene expression data with 14846 genes, we selected 1682 genes with significant expression variances for network inference. With the selected gene expression data, RSNET method inferred a densely connected network with 1530 genes and 14446 edges. After deleting the edges with low correlation strengths, a core network with 313 genes and 1425 edges was the final network inferred. The Gene Ontology (GO) analysis for these genes and the comparison analysis with differential expression were implemented. Additional file 2: Table S2 shows the function of these identified genes.

**Fig. 6:**
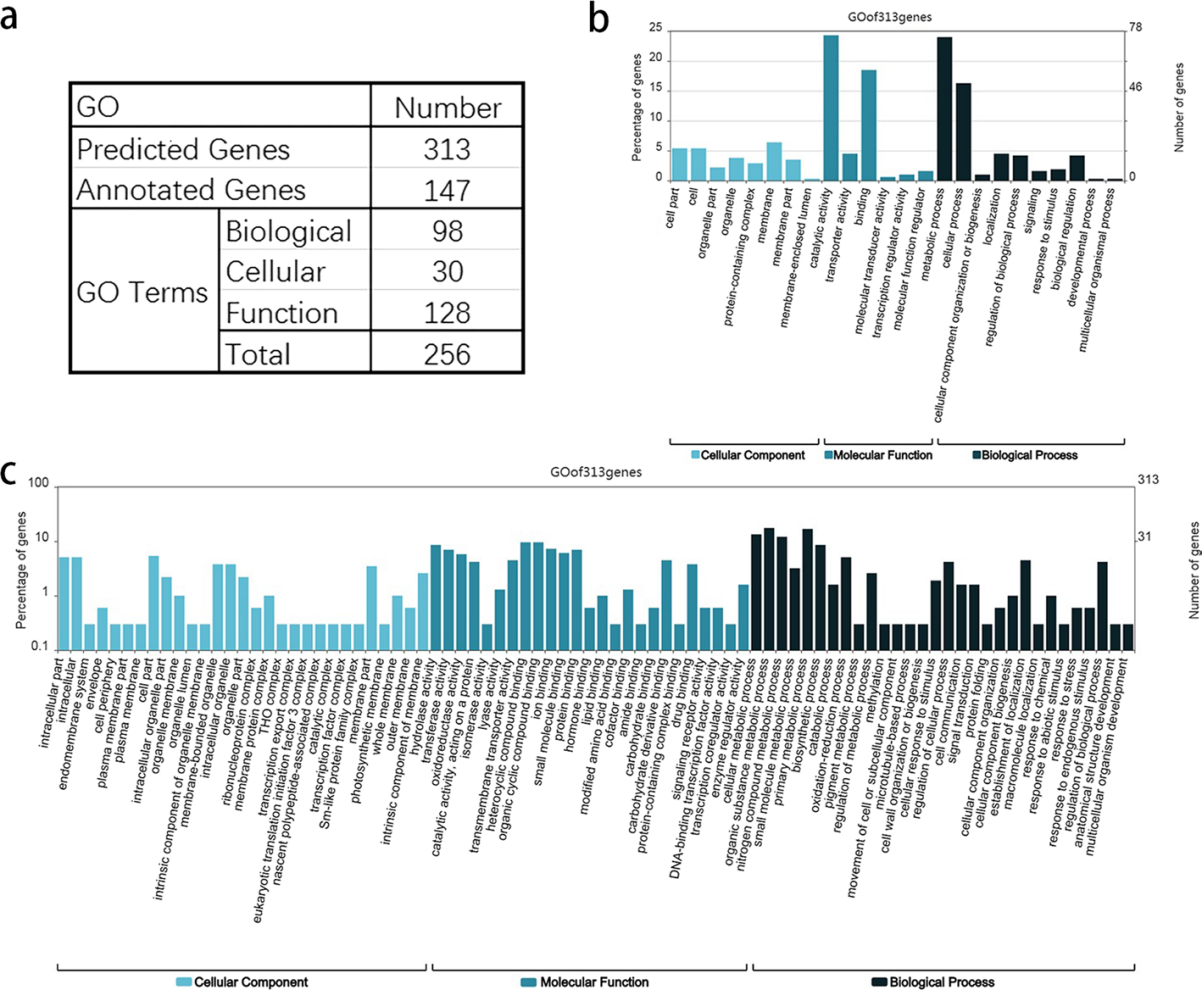
The GO analysis confirmed the genes predicted correlated with fruit development. **a** Table for the result of GO analysis including the number of genes involved in different GO terms. **b** Hierarchical relationship of the gene set in second level of GO items. **c** Hierarchical relationship of gene set in third level of GO items

To process GO analysis, all the nucleotide sequences from NCBI database in FASTA format were downloaded firstly and then annotate the sequences using the 'Blastn' module in Blast2GO. After the analysis of 'blast', 'mapping', 'annotation' and 'interproscan', a hierarchical relationship of GO items (http://geneontology.org/) was achieved. With above GO items, the web tool WEGO2.0 (http://wego.genomics.org.cn/) was used for the visualization. Figure 6 shows the result of GO analysis for the genes identified. Out of 313 core genes, 147 genes were annotated and divided into three basic parts in GO first-level items (Additional file 3: Table S3). There are 98 items in biological process part, 30 items in cellular component and 128 items in molecular function part (Fig. 6a). To show the hierarchical relationship for the gene set, the second and third levels of GO items were provided separately (Fig. 6b, c). Listed in first and third places of the columns, two items catalytic activity (GO:0003824) and binding (GO:0005488) reveal that these genes are involved in some catalytic reactions and molecule activities, such as redox reactions, hydrolysis reaction, ion binding, organic cyclic compound binding, etc. Another two items metabolic process (GO:0008152) and cellular process (GO:0009987), listed in second and forth places, indicate that the genes regulate some metabolism related biological progresses. All items above confirm that the gene set identified by RSNET method are highly correlated with fruit developmental progress.

**Fig. 7:**
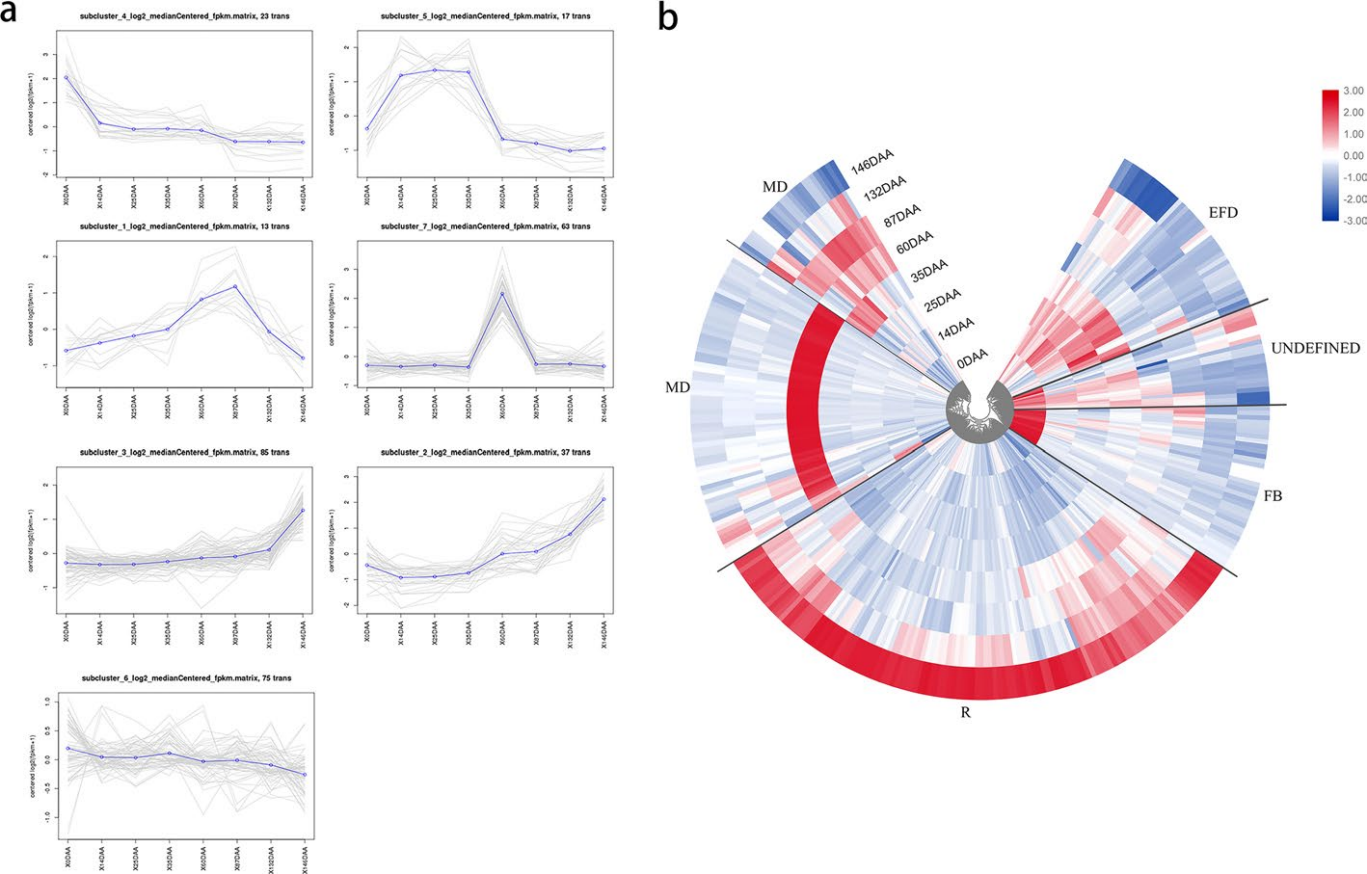
The clustering analysis for dynamical gene expression confirmed the genes predicted correlated with fruit development. **a** Seven sub-clusters of genes with dynamical changes during eight fruit developmental stages. **b** The heat-map of clustering of genes in four different fruit developmental stages

To explore whether the genes identified by RSNET method correlate with fruit development, we analyzed the dynamical changes of their expression during the stages from floral bud to ripe fruit. We clustered the 313 genes into seven sub-clusters with clustering tool. Among of them, six sub-clusters are matched with the four plant physiological processes, i.e. floral bud/bloom (FB), early fruit development (EDF), mid-development (MD), and ripening (R) (Fig. 7a). This result showed that the sub-cluster 4 matched FB, the sub-cluster 5 matched EDF, the sub-clusters 1 and 7 matched MD and sub-clusters 2 & 3 matched R exactly (Fig. 7b). Our analysis provides a gene list with significance for fruit development. Among of these genes in the list, 30 genes are highly related ones and 283 genes are related ones. Compared to previous analysis by ANOVA method which selected 1955 genes, RSNET method show the superiority in smaller gene size for showing the similar dynamical change with fruit development. With fewer genes, RSNET method significantly caught the dynamical changing during fruit development. The result shows two advantages of RSNET method in network inference. Firstly, RSNET method can identify the direct causal genes by filtering out the indirect and noisy genes. Secondly, RSNET method can identify significant genes but not a random selection from the whole genes.

## Methods

### Mutual information between gene pairs

The dependency between a gene pair can be measured by computing mutual information (MI) of two gene expression vectors. For the advantage of nonlinear relationship measurement, mutual information has been widely used. For gene pair *A* and *B*, their mutual information (MI) can be described as [33]

1 $$MI(A,B) = - \sum\limits_{a \in A,\;b \in B} {f(a,b)\log \frac{f(a,b)}{{f(a)f(b)}}} .$$

With mathematical analysis, above formula can be commutated by [33]

2 $$MI(A,B) = \frac{1}{2}\log \frac{|M(A)| \cdot |M(B)|}{{|M(A,B)|}},$$

where *M* is covariance matrix and |*M*| is the determinant of *M*. In particular, *MI*(*A,B*) = 0 represents that genes *A* and *B* are independent.

In the first step of the proposed method, mutual information will be used to select the putative regulators from the global candidate genes for a given target gene.

### Redundancy silencing and network enhancement technique

To quantitatively describe a gene regulatory network for the transcription procedure from DNA to RNA, a mathematical model involving transcription factors and target gene should be built [45, 54]. Among the reasonable models, regression model is the most popular one for its advantage of dynamic description of transcription. In this work, we provided an update model to silence the redundant regulations and enhance the high-confident edges. The redundancy silencing is implemented by the following recursive optimizations with update results until there is no change for the result.

3 $$\tilde{\beta } = \mathop {\min }\limits_{\beta } \left| {y - \beta X} \right| + \lambda \left| \beta \right| + \gamma \left| {\hat{\beta } \otimes \beta } \right|.$$

where $y,X$ and $\beta$ represent target gene, TFs, and regulatory strengths respectively. $\hat{\beta }$ is the network enhancement items with 0 or 1. $\lambda$ and $\gamma$ are parameters to balance the error and ensure the network sparseness respectively. The operator $\otimes$ is the Hadamard product. The parameter $\hat{\beta }$ will be estimated by mutual information firstly and then updated by optimizations [55]. As a linear programming model, Eq. (3) can be resolved for the estimation of $\tilde{\beta }$ which will be taken as regulatory strengths of network.

### Pseudo-code of RSNET algorithm

As follows is the pseudo-code of RSNET algorithm.
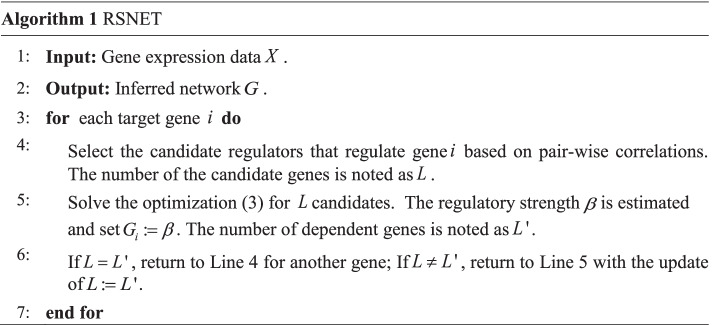


### Benchmark networks and evaluation

To evaluate the efficiency of network inference, RSNET algorithm was implemented on synthetic and experimental gene expression data. For synthetic data, the method was assessed by using simulation study and the widely used dataset from DREAM challenge [7, 56]. For experimentally measured data, we used the gold-standard *Escherichia coli* data [49]. The experimental gene expression data was collected and preprocessed from the dataset for *Escherichia coli* [50]. There are 160 regulators, 1258 targets and 2675 links in the experimentally verified network. As a case study, we also used RSNET method to reconstruct functional GRN for apple fruit development from gene expression data [53].

To show the superiority of RSNET method, the comparisons with some popular methods including LASSO [36], LP [57], RO [45], GENIE3 [58], ARACNE and NARROMI [45] were implemented. LASSO represents the network inference based on regression model. LP represents the network inference method based on linear programming. RO represents the network inference based on recursive optimization method. GENIE3 represents the network inference method with random forest. ARACNE represents network inference based on mutual information (MI). NARROMI represents the network inference based on a noise and redundancy reduction strategy.

To assess the performance of these compared methods, we use some standard quantitative measures to score the prediction results. For example, accuracy (ACC), Matthews Coefficient Constant (MCC), positive predictive value (PPV), false positive rate (FPR) and true positive rate (TPR). We also plot the receiver operating characteristic (ROC) curve and calculate the area under ROC curve (AUC) based on above measures to show the performance.

## Discussion

In this work, we developed a feature selection method based on a redundancy silencing and network enhancement technique to address the issue that numerous indirect interactions inherited in the predictions. In the proposed method, highly dependent nodes are constrained in the model as network enhancement items to enhance real interactions, and dimensionality of putative interaction is reduced adaptively to remove weak and indirect connections.

There are some advantages of RSNET method in network inference. Firstly, it improves the accuracy of network inference through a redundancy silencing and network enhancement technique. The developed algorithm has the ability of filtering weak interactions, keeping high interactions, and silencing indirect interactions. In the initial step, MI filters out the noisy interactions by detecting low-, mid- and high- dependences. Then the high-dependence regulations are constrained in the model to keep these interactions in the result. The recursive optimizations with update candidates reduce the indirect interactions step by step and keep the direct interactions in final prediction. Secondly, the network inferred by RSNET method is a directed network. This is different from mutual information (MI)-based methods which cannot detect the directions of network. Thirdly, the technique combining both linear and nonlinear interactions overcomes the drawback of linear or nonlinear methods. As a technique for parameter estimation of regression and feature selection, this model can also be used for data mining in other areas.

## Conclusion

In reconstruction of GRNs, distinguishing the direct interactions from the indirect ones is an important challenge because of the notoriousness of the inference methods with the indirect interactions inherited in the network. In this study, we present a redundancy silencing and network enhancement technique-based network inference method named RSNET. In the proposed method, the redundant interactions including weak and indirect connections are silenced by recursive optimization adaptively. While the highly confident correlated regulators are constrained to improve the true positive rate of prediction. The results on gold-standard networks including simulation study, DREAM challenge dataset and Escherichia coli network show the good performance of RSNET method. The case study for constructing apple fruit ripening GRN show that RSNET method can construct function-specific GRNs. This study provides a useful bioinformatics tool for inferring clean GRN from gene expression data.

## Supplementary Information

**Additional file 1: Table S1**. The results on DREAM networks of *E.coli* 1, *E.coli* 2, Yeast 2 and Yeast 3.

**Additional file 2: Table S2**. The function of the identified genes for apple fruit development.

**Additional file 3: Table S3**. The GO first-level items of the identified genes for apple fruit development.

## Data Availability

The RSNET software and related data are freely accessible at https://github.com/zhanglab-wbgcas/rsnet. The raw data of apple gene expression analyzed in this study are available at https://www.ncbi.nlm.nih.gov/pmc/articles/PMC2287172/bin/1471-2229-8-16-S1.xls.

## Abbreviations

GRN: Gene regulatory network
MI: Mutual information
TF: Transcription factor
TG: Target gene
GO: Gene ontology
EDF: Early fruit development
MD: Mid-development
FB: Full bloom
R: Ripening
TPR: True positive rate
FPR: False positive rate
PPV: Positive predictive value
ACC: Accuracy
MCC: Matthews correlation coefficient
ROC: Receiver operating characteristic
AUC: Area under ROC curve

## Availability and requirements

Project name: rsnet. Project home page: https://github.com/zhanglab-wbgcas/rsnet. Operating system: Windows. Programming language: MATLAB. Other requirements: MATLAB 7.0 or higher. License: MATLAB. Any restrictions to use by non-academics: None.
