# Supplementary material for: RSNET: inferring gene regulatory networks by a redundancy silencing and network enhancement technique
Source: BMC Bioinformatics. 2022 May 6;23:165. doi: 10.1186/s12859-022-04696-w (PMC9074326; doi:10.1186/s12859-022-04696-w)
Supplement: Supplementary file 1 — Additional file 1: Table S1. The results on DREAM networks of E.coli 1, E.coli 2, Yeast 2 and Yeast 3. [file 12859_2022_4696_MOESM1_ESM.docx]

#### Supplementary Table S1. Results on DREAM networks of E.coli 1, E.coli 2, Yeast 2 and Yeast 3 with scales 10, 50 and 100.

#### (1) Network of E.coli 1.

| Approach | FPR | TPR | ACC | PPV | MCC | AUC |
| --- | --- | --- | --- | --- | --- | --- |
| Scale 10 |  |  |  |  |  |  |
| LASSO | 0.7215 | 0.8181 | 0.3444 | 0.1363 | 0.0715 | 0.5293 |
| LP | 0.2278 | 0.1818 | 0.7000 | 0.1000 | -0.0362 | 0.5777 |
| RO | 0.3670 | 0.2727 | 0.5888 | 0.0937 | -0.0645 | 0.6053 |
| GENIE3 | 0.2151 | 0.5454 | 0.7555 | 0.2608 | 0.2480 | 0.6997 |
| ARACNE | 0.2278 | 0.5454 | 0.7444 | 0.2500 | 0.2352 | 0.7371 |
| NARROMI | 0.2278 | 0.5454 | 0.7444 | 0.2500 | 0.2352 | 0.7365 |
| **RSNET** | **0.2151** | **0.4545** | **0.7444** | **0.2272** | **0.1824** | **0.7359** |
| Scale 50 |  |  |  |  |  |  |
| LASSO | 0.1214 | 0.3548 | 0.8653 | 0.0705 | 0.1099 | 0.6927 |
| LP | 0.0820 | 0.4677 | 0.9065 | 0.1288 | 0.2097 | 0.6859 |
| RO | 0.0946 | 0.5161 | 0.8955 | 0.1240 | 0.2156 | 0.7358 |
| GENIE3 | 0.0866 | 0.4193 | 0.9008 | 0.1115 | 0.1781 | 0.7550 |
| ARACNE | 0.1210 | 0.6935 | 0.8742 | 0.1295 | 0.2627 | 0.8355 |
| NARROMI | 0.0770 | 0.6451 | 0.9159 | 0.1785 | 0.3095 | 0.8455 |
| **RSNET** | **0.0678** | **0.6451** | **0.9248** | **0.1980** | **0.3296** | **0.8475** |
| Scale 100 |  |  |  |  |  |  |
| LASSO | 0.0652 | 0.2880 | 0.9265 | 0.0534 | 0.0987 | 0.7220 |
| LP | 0.0500 | 0.3840 | 0.9428 | 0.0893 | 0.1646 | 0.6613 |
| RO | 0.0802 | 0.4480 | 0.9138 | 0.0666 | 0.1473 | 0.7439 |
| GENIE3 | 0.0186 | 0.3200 | 0.9730 | 0.1801 | 0.2272 | 0.8531 |
| ARACNE | 0.0574 | 0.6080 | 0.9382 | 0.1191 | 0.2503 | 0.8197 |
| NARROMI | 0.0368 | 0.5600 | 0.9580 | 0.1627 | 0.2865 | 0.8559 |
| **RSNET** | **0.0376** | **0.6080** | **0.9578** | **0.1711** | **0.3076** | **0.8586** |

#### (2) Network of E.coli 2.

| Approach | FPR | TPR | ACC | PPV | MCC | AUC |
| --- | --- | --- | --- | --- | --- | --- |
| Scale 10 |  |  |  |  |  |  |
| LASSO | 0.6400 | 0.6666 | 0.4111 | 0.1724 | 0.0207 | 0.5644 |
| LP | 0.3066 | 0.2666 | 0.6222 | 0.1481 | -0.0325 | 0.5920 |
| RO | 0.2800 | 0.4666 | 0.6777 | 0.2500 | 0.1502 | 0.5316 |
| GENIE3 | 0.1733 | 0.4000 | 0.7555 | 0.3157 | 0.2069 | 0.6844 |
| ARACNE | 0.3066 | 0.6000 | 0.6777 | 0.2812 | 0.2283 | 0.6760 |
| NARROMI | 0.3066 | 0.6000 | 0.6777 | 0.2812 | 0.2283 | 0.6787 |
| **RSNET** | **0.2000** | **0.5333** | **0.7555** | **0.3478** | **0.2848** | **0.6996** |
| Scale 50 |  |  |  |  |  |  |
| LASSO | 0.1106 | 0.2560 | 0.8681 | 0.0742 | 0.0818 | 0.6739 |
| LP | 0.0945 | 0.3414 | 0.8865 | 0.1111 | 0.1461 | 0.6552 |
| RO | 0.1321 | 0.4512 | 0.8538 | 0.1057 | 0.1639 | 0.7065 |
| GENIE3 | 0.0793 | 0.5121 | 0.9069 | 0.1826 | 0.2668 | 0.7816 |
| ARACNE | 0.1769 | 0.6951 | 0.8187 | 0.1197 | 0.2355 | 0.8225 |
| NARROMI | 0.0983 | 0.5975 | 0.8914 | 0.1737 | 0.2813 | 0.8262 |
| **RSNET** | **0.0899** | **0.5975** | **0.8995** | **0.1870** | **0.2954** | **0.8265** |
| Scale 100 |  |  |  |  |  |  |
| LASSO | 0.0630 | 0.2268 | 0.9283 | 0.0419 | 0.0723 | 0.6934 |
| LP | 0.0560 | 0.4285 | 0.9377 | 0.0851 | 0.1702 | 0.6953 |
| RO | 0.0846 | 0.3445 | 0.9084 | 0.0471 | 0.1000 | 0.6806 |
| GENIE3 | 0.0239 | 0.4369 | 0.9695 | 0.1818 | 0.2687 | 0.9016 |
| ARACNE | 0.0879 | 0.7731 | 0.9104 | 0.0966 | 0.2532 | 0.8918 |
| NARROMI | 0.0594 | 0.7563 | 0.9383 | 0.1341 | 0.3021 | 0.9027 |
| **RSNET** | **0.0590** | **0.7478** | **0.9385** | **0.1334** | **0.2994** | **0.9021** |

#### (3) Network of Yeast 2

| Approach | FPR | TPR | ACC | PPV | MCC | AUC |
| --- | --- | --- | --- | --- | --- | --- |
| Scale 10 |  |  |  |  |  |  |
| LASSO | 0.6307 | 0.6000 | 0.4333 | 0.2678 | -0.0284 | 0.5052 |
| LP | 0.1538 | 0.2400 | 0.6777 | 0.3750 | 0.1009 | 0.5194 |
| RO | 0.2461 | 0.2800 | 0.6222 | 0.3043 | 0.0347 | 0.4418 |
| GENIE3 | 0.1230 | 0.2400 | 0.7000 | 0.4285 | 0.1444 | 0.5618 |
| ARACNE | 0.1384 | 0.2000 | 0.6777 | 0.3571 | 0.0760 | 0.5455 |
| NARROMI | 0.1384 | 0.2000 | 0.6777 | 0.3571 | 0.0760 | 0.5449 |
| **RSNET** | **0.1384** | **0.2400** | **0.6888** | **0.4000** | **0.1220** | **0.5831** |
| Scale 50 |  |  |  |  |  |  |
| LASSO | 0.1301 | 0.2125 | 0.8269 | 0.1024 | 0.0594 | 0.6219 |
| LP | 0.0868 | 0.2750 | 0.8714 | 0.1810 | 0.1554 | 0.5756 |
| RO | 0.1484 | 0.3250 | 0.8171 | 0.1326 | 0.1189 | 0.6019 |
| GENIE3 | 0.1218 | 0.3312 | 0.8775 | 0.2154 | 0.2030 | 0.6886 |
| ARACNE | 0.0842 | 0.3937 | 0.8465 | 0.1842 | 0.1938 | 0.7133 |
| NARROMI | 0.0812 | 0.3562 | 0.8820 | 0.2345 | 0.2273 | 0.7202 |
| **RSNET** | **0.0733** | **0.3312** | **0.8877** | **0.2398** | **0.2224** | **0.7188** |
| Scale 100 |  |  |  |  |  |  |
| LASSO | 0.0492 | 0.1208 | 0.9181 | 0.0912 | 0.0626 | 0.6239 |
| LP | 0.0517 | 0.3341 | 0.9241 | 0.2090 | 0.2261 | 0.6378 |
| RO | 0.1043 | 0.3393 | 0.8738 | 0.1174 | 0.1439 | 0.6398 |
| GENIE3 | 0.0246 | 0.2262 | 0.9459 | 0.2732 | 0.2208 | 0.7245 |
| ARACNE | 0.0527 | 0.3393 | 0.9233 | 0.2082 | 0.2274 | 0.7629 |
| NARROMI | 0.0293 | 0.2956 | 0.9441 | 0.2918 | 0.2646 | 0.7647 |
| **RSNET** | **0.0311** | **0.3059** | **0.9428** | **0.2867** | **0.2664** | **0.7639** |

#### (4) Network of Yeast 3

| Approach | FPR | TPR | ACC | PPV | MCC | AUC |
| --- | --- | --- | --- | --- | --- | --- |
| Scale 10 |  |  |  |  |  |  |
| LASSO | 0.8529 | 0.8636 | 0.3222 | 0.2467 | 0.0130 | 0.4619 |
| LP | 0.2058 | 0.1363 | 0.6333 | 0.1764 | -0.0763 | 0.4452 |
| RO | 0.2647 | 0.2727 | 0.6222 | 0.2500 | 0.0077 | 0.5140 |
| GENIE3 | 0.1470 | 0.3636 | 0.7333 | 0.4444 | 0.2326 | 0.6557 |
| ARACNE | 0.5294 | 0.8181 | 0.5555 | 0.3333 | 0.2533 | 0.6965 |
| NARROMI | 0.5294 | 0.8181 | 0.5555 | 0.3333 | 0.2533 | 0.6975 |
| **RSNET** | **0.2647** | **0.5909** | **0.7000** | **0.4193** | **0.2950** | **0.7079** |
| Scale 50 |  |  |  |  |  |  |
| LASSO | 0.1286 | 0.1849 | 0.8228 | 0.0984 | 0.0425 | 0.5775 |
| LP | 0.1023 | 0.2427 | 0.8514 | 0.1527 | 0.1139 | 0.5518 |
| RO | 0.1567 | 0.3757 | 0.8102 | 0.1540 | 0.1485 | 0.6183 |
| GENIE3 | 0.0614 | 0.2774 | 0.8918 | 0.2553 | 0.2078 | 0.6574 |
| ARACNE | 0.1277 | 0.3294 | 0.8338 | 0.1637 | 0.1479 | 0.6412 |
| NARROMI | 0.0790 | 0.2601 | 0.8742 | 0.2000 | 0.1606 | 0.6438 |
| **RSNET** | **0.0645** | **0.2485** | **0.8869** | **0.2263** | **0.1762** | **0.6427** |
| Scale 100 |  |  |  |  |  |  |
| LASSO | 0.0546 | 0.1270 | 0.8997 | 0.1204 | 0.0706 | 0.6216 |
| LP | 0.0519 | 0.2359 | 0.9083 | 0.2110 | 0.1745 | 0.6025 |
| RO | 0.0982 | 0.3139 | 0.8689 | 0.1584 | 0.1578 | 0.6415 |
| GENIE3 | 0.0226 | 0.1578 | 0.9317 | 0.2909 | 0.1811 | 0.6602 |
| ARACNE | 0.0393 | 0.2141 | 0.9190 | 0.2427 | 0.1854 | 0.6949 |
| NARROMI | 0.0243 | 0.1705 | 0.9308 | 0.2919 | 0.1889 | 0.6960 |
| **RSNET** | **0.0279** | **0.1996** | **0.9290** | **0.2964** | **0.2072** | **0.6957** |
