# Supplementary material for: RSNET: inferring gene regulatory networks by a redundancy silencing and network enhancement technique
Source: BMC Bioinformatics. 2022 May 6;23:165. doi: 10.1186/s12859-022-04696-w (PMC9074326; doi:10.1186/s12859-022-04696-w)
Supplement: Supplementary file 2 — Additional file 2: Table S2. The function of the identified genes for apple fruit development. [file 12859_2022_4696_MOESM2_ESM.docx]

**Supplementary Table S2.** The function of the identified genes for apple fruit development.

| No. | Gene name | Type | Function |
| --- | --- | --- | --- |
| 1 | CN936403 | Cellcycle | methyladenine glycosylase family protein |
| 2 | EB107042 | Cellcycle | CDKB1;2 cell division control protein |
| 3 | CN929052 | Cellcycle | dimethyladenosine transferase |
| 4 | CN862228 | Cellcycle | zinc carboxypeptidase family protein |
| 5 | CN864463 | Cellcycle | protein phosphatase 2A-associated 46 kDa protein |
| 6 | EB119954 | Cellcycle | heavy-metal-associated domain-containing protein |
| 7 | CN937737 | Cellcycle | zinc finger (C3HC4-type RING finger) family protein |
| 8 | CN888558 | Cellcycle | DNAJ heat shock family protein |
| 9 | CN938500 | Cellcycle | replication protein, putative |
| 10 | CN876164 | Cellcycle | nucleoside diphosphate kinase 3, mitochondrial (NDK3) |
| 11 | EG631233 | Cellcycle | myb family transcription factor (MYB83) |
| 12 | CN912925 | Cellcycle | dynamin-like protein 6 (ADL6) |
| 13 | EB151655 | Cellcycle | EMB1135 DNA-binding protein, putative |
| 14 | CN908171 | Cellcycle | guanylate kinase 2 (GK-2) |
| 15 | CN914773 | Cellcycle | ubiquitin activating enzyme 1 (UBA1) |
| 16 | CN910366 | Cellcycle | E2F-like repressor E2L3 (E2L3) |
| 17 | CN917058 | Cellcycle | transducin family protein/WD-40 repeat family protein |
| 18 | EB109178 | Cellcycle | UV hypersensitive protein (UVH3) |
| 19 | CN931474 | Cellcycle | ATP-dependent DNA helicase, putative |
| 20 | CN932236 | Cellcycle | methyladenine glycosylase family protein |
| ... | ... | ... | ... |
