# Supplementary material for: RSNET: inferring gene regulatory networks by a redundancy silencing and network enhancement technique
Source: BMC Bioinformatics. 2022 May 6;23:165. doi: 10.1186/s12859-022-04696-w (PMC9074326; doi:10.1186/s12859-022-04696-w)
Supplement: Supplementary file 3 — Additional file 3: Table S3. The GO first-level items of the identified genes for apple fruit development. [file 12859_2022_4696_MOESM3_ESM.docx]

#### Supplementary Table S2. The GO first-level items of the identified genes for apple fruit development.

| No. | Gene | Type | Descriptions | Edges | Map | Rank |
| --- | --- | --- | --- | --- | --- | --- |
| 1 | CN864463 | Cellcycle | protein phosphatase 2A-associated 46 kDa protein | 2 | '29.4' | 4 |
| 2 | CN886787 | Cellcycle | similar to tetratricopeptide repeat (TPR)-containing | 17 | '35.1.5' | 7 |
| 3 | CN914478 | Cellcycle | 0heat shock protein 81-1 (HSP81-1) | 1 | '20.2.1' | 6 |
| 4 | EB124712 | Arb | Yabby | 8 | '27.3.10' | 7 |
| 5 | EB131105 | tomato | Photosystem I reaction center subunit psaK 3.00E-10 | 2 | '1.1.2.2' | 5 |
| 6 | CN943669 | tomato | Plasma membrane intrinsic protein 5.00E-35 | 1 | '34.19.1' | 1 |
| 7 | EE663684 | tomato | 17.6 kDa class I heat shock protein 2.00E-12 | 1 | '20.2.1' | 1 |
| 8 | CN894718 | tomato | aspartyl protease family protein 9.00E-16 | 1 | '29.5.4' | 3 |
| 9 | EB152301 | tomato | 14-3-3 protein GF14 upsilon (GRF5) 2.00E-12 | 2 | '30.7' | 3 |
| 10 | EB128426 | tomato | vacuolar processing enzyme-1b 3.00E-12 | 2 | '29.3.4.3' | 2 |
| 11 | EE663883 | tomato | Expressed protein 9.00E-12 | 1 | '35.2' | 2 |
| 12 | CN898201 | tomato | short chain dehydrogenase/reductase family 3.00E-08 | 8 | '26.22' | 2 |
| 13 | EG631323 | tomato | N-benzoyltransferase protein 4.00E-06 | 1 | '20.1' | 2 |
| 14 | CN870279 | Fuji | 16.9 kDa class I heat shock protein | 1 | '20.2.1' | 1 |
| 15 | EG631337 | Fuji | class I heat shock protein | 1 | '20.2.1' | 1 |
| 16 | CN862135 | ethy | Hypothetical protein | 7 | '35.2' | 3 |
| 17 | EB114937 | ethy | β-glucosidase precursor | 22 | '10.6.2' | 2 |
| 18 | CN862240 | ethy | Transaldolase ToTAL2 | 16 | '7.2.2' | 3 |
| 19 | CN863631 | ethy | Sugar transporter | 2 | '34.2' | 3 |
| 20 | EB117418 | ethy | (1–4)-β-mannan endohydrolase | 1 | '10.6.2' | 2 |
| 21 | EB139752 | ethy | Seed storage/lipid transfer protein | 10 | '26.21' | 3 |
| 22 | CN901620 | ethy | Hypothetical protein | 1 | '33.2' | 3 |
| 23 | EG631195 | ethy | Transferase family protein | 16 | '35.1' | 2 |
| 24 | EG631213 | ethy | β-amylase | 1 | '2.2.2.1.2' | 2 |
| 25 | CN912930 | ethy | Pentatricopeptide repeat protein | 17 | '35.1.5' | 2 |
| 26 | CN917441 | ethy | Dormancy/auxin associated | 1 | '33.99' | 3 |
| 27 | CN915067 | ethy | Sugar transporter family protein | 2 | '34.2' | 2 |
| 28 | EE663891 | ethy | Polygalacturonase | 6 | '10.6.3' | 2 |
| 29 | CN941807 | ethy | DEAD box RNA helicase | 1 | '28.1' | 3 |
| 30 | CN883038 | ethy | Hypothetical protein | 1 | '35.2' | 3 |
